# Path dependence in social and psychological risk factors for dementia

**DOI:** 10.1590/S1980-57642011DN05010002

**Published:** 2011

**Authors:** Hiroko Matsuoka, Hidehiko Yamaguchi

**Affiliations:** 1 MA, School of Nursing & Health, Aichi Prefectural University, Nagoya, Japan.; 2 MA, Volunteer Group “Transpacífico”, Mexico City, Mexico.

**Keywords:** Alzheimer’s disease, dementia, risk factors, prevention, developing countries

## Abstract

This article focuses on social and psychological risk factors for Alzheimer’s disease, dementia, and cognitive impairment and presents some key points for prevention in developing countries based on previous studies, a social science theory, and our preliminary survey. Previous population-based studies found that educational and occupational attainment, income, participation in social and mental activities, and psychological distress were associated with dementia risk. According to the theory of path dependence, earlier factors largely determine successive ones, where education is one of these early experiences in life. Our preliminary survey suggested that education sets a path that several psychosocial risk factors are dependent on. The expansion of basic education is indispensable. Resources for prevention should be concentrated on individuals with a low level of education. In order to break from a path creating self-reinforcement of risk factors, it is necessary to implement early and active interventions.

## Abstract (pt)

Este artigo foca nos fatores de risco social e psicológico para doença de Alzheimer, demência e comprometimento cognitivo e apresenta alguns pontos chave para prevenção em países em desenvolvimento baseado em estudos prévios, uma teoria da ciência social e nossa pesquisa preliminar. Estudos populacionais prévios encontraram que educação, ocupação, ganhos, participação em atividades mentais e sociais, e estresse psicológico associaram-se com o risco. De acordo com a teoria da dependência da trajetória, fatores precoces determinam os fatores sucessivos e nós podemos afirmar que educação é uma das experiências mais precoces na vida. Nossa pesquisa preliminar sugere que a educação determina uma via em que vários fatores de risco psicossociais são dependentes. A ampliação da educação básica é indispensável. Fontes para prevenção devem ser concentradas nos indivíduos com baixo nível educacional. A fim de desviar da via que cria um auto-reforço de fatores de risco, é necessário implementar intervenções precoces e ativas.

A remarkable demographic transition is underway. Globally, the number of persons over 60 years of age is set to increase from about 600 million in 2000 to almost 2 billion by 2050, and this increase will be greatest and most rapid in developing countries.^1^ The incidence of dementia and Alzheimer’s disease (AD) rises exponentially with age.^2^ Therefore, the number of individuals with dementia will increase rapidly in developing countries.

Developing countries face the challenge of simultaneous development and population aging.^1^ Although dementia patients need specialized medical and long-term care, the resources for such care are very limited in most cases. Also, developing countries have to direct their public resources primarily into economic growth. Furthermore, the capacity of families in these countries to support older people will decrease with a lower birthrate and rapid industrialization and urbanization. This may create a critical situation if older people with dementia cannot rely on support from their families or on social security. Therefore, effective and efficient policies for preventing dementia are crucial until a specific remedy for the disease is developed and popularized.

Previous population-based studies have identified several social and psychological risk factors for AD, dementia, and cognitive impairment including educational and occupational attainment, income, participation in social and mental activities, and psychological distress. This study presents some key points for effective and efficient prevention that are based on these findings, on a basic theory of social science, and suggestions drawn from our preliminary survey.

## Psychosocial risk factors

**Education** – Many studies have considered education to be an important risk factor for dementia or cognitive decline, using years of schooling as a measure of educational level. A New York study found that lower educational level was associated with a higher risk of dementia,^3^ and similarly a study in Italy pointed out the association between no education and the prevalence of dementia.^4^ According to the results of the Kungsholmen Project in Stockholm, a low level of education was associated with an increased risk of AD or dementia.^5,6^ Data from the Assets and Health Dynamics of the Oldest Old (AHEAD) study in the U.S. showed a relationship between higher education and better maintenance of cognitive function^7^ and indicated that those with low levels of education were more likely to become cognitively impaired.^8^ A Kuopio study in Finland associated educational contribution with levels of cognitive functioning in late middle age,^9^ while an analysis of the data from the Canadian Study of Health and Aging (CSHA) concluded that there was an association between low education level and an elevated risk of AD and vascular dementia.^10^

Studies in developing countries have shown the same trend. A survey in China found that receiving some education was associated with a lower probability of suffering from cognitive impairment in old age.^11^ Results of the SABE Project, a study conducted in seven Latin American and Caribbean capital cities, indicated that illiteracy was associated with higher chances of cognitive impairment after the age of 60 years.^12^ Data from eight studies in six Latin American countries revealed that the prevalence of dementia in illiterates was two times higher than in non-illiterates and showed that the illiteracy rate among the elderly in the region was 9.3%.^13^

**Occupation** – Some studies have regarded occupation as an independent risk factor. A New York study argued that lower occupational attainment, excluding managerial and professional positions, was associated with a higher risk of dementia.^3^ In a sample of Australian men, realistic occupations, including trade, technical, and certain service occupations, were related to poorer cognitive performance and a higher prevalence of dementia.^14^ In a study in Taiwan, non-intellectual workers and housekeepers were at higher risks of cognitive decline.^15^ Among the respondents in the SABE Project, those who worked in unskilled occupations exhibited a higher prevalence of cognitive impairment.^12^ Findings of a Canadian study using data from the CSHA, highlighted that highly complex work appeared to be associated with a reduced risk of dementia.^16^

**Income** – Several studies have focused on economic variables such as income. In a sample of eastern Finnish men, better cognitive performance was associated with higher income.^9^ A Leiden study in the Netherlands found dementia to be more prevalent in those on low income.^17^ Another Finnish survey, carried out in Kuopio and Joensuu, indicated that low income level at old age was related to an increased risk of dementia,^18^ and the results of the SABE Project revealed that having insufficient income in old age was associated with higher chance of cognitive impairment.^12^

**Participation in social and mental activities** – Some previous studies have focused not only on life course, but also on lifestyle, especially participation in activities. A New Haven study in the U.S. indicated that social disengagement was significantly associated with the incidence of cognitive decline,^19^ and a study by the Rush Alzheimer’s Disease Center (RADC) in the U.S. suggested that premorbid reading activity had protective effects against AD.^20^ A study in New York found that the risk of dementia was decreased in subjects practicing a high amount of leisure activities, especially those involving intellectual factors,^21^ whereas the Kungsholmen Project found that the incidence of dementia decreased with increasing frequency of participation in mental, social, or productive activities.^22^ The U.S. Religious Order Study suggested that frequent participation in cognitively stimulating activities in old age was associated with a reduced risk of AD,^23^ and the Bronx Aging Study in the U.S. also found that participation in cognitive activities was associated with a reduced risk of AD, vascular dementia, and mixed dementia.^24^ In a random sample of Taiwanese individuals, participation in social activities outside the family was significantly associated with a reduced risk of cognitive impairment.^25^

**Social risk factors and dementia** – It is known that there is a significant gap between the clinical presentation of dementia and its pathological development. The cognitive reserve hypothesis explains this gap. This reserve allows an individual to cope longer before dementia is clinically expressed. Genetic predisposition as well as life experience, such as education and occupation, and lifestyle in old age, including participation in activities, might provide this cognitive reserve.^26-30^ Therefore, we can postulate that it may be possible to prevent the disease clinically by creating a favorable environment.

Higher income in old age allows greater exposure to more stimulating environments through leisure activities. It also guarantees greater access to quality medical care in earlier stages of disease and therefore to better health maintenance, especially in developing countries.

**Psychological distress** – Many researchers believe psychological distress, particularly depression and loneliness, to be closely related to risk of dementia. A Dutch survey concluded that there was a significant relationship between old-age depression and subsequent dementia,^31^ and another study in the Netherlands, using samples from Amsterdam, showed that depression increased the risk of AD and cognitive decline, respectively, although only among those with higher levels of education.^32^ The Multi-institutional Research in Alzheimer’s Genetic Epidemiology (MIRAGE) Study suggested that depression symptoms were a risk factor for later development of AD.^33^ The Religious Order Study found that the tendency to experience psychological distress was related to risk of clinical AD, independent of its pathology.^34^ According to the results of a census in neighborhoods of Chicago, levels of depressive symptoms were related to the rate of global cognitive decline.^35^ A study in the Kungsholmen Project found that loneliness and low mood were associated with an increased risk of dementia,^36^ and research in the Rush Memory and Aging Project (RMAP) in the U.S. discovered that a higher level of loneliness was strongly associated with a higher likelihood of developing AD.^37^ A study in Spain indicated that depression was an independent risk factor for dementia, AD, and vascular dementia.^38^

Psychological distress creates an inactive and socially isolated lifestyle, and this situation tends to elevate the risk of cognitive impairment. On the other hand, such distress is also a reaction to perceived cognitive decline. We can presume that psychological distress, such as depression, disinterest, agitation, apathy, anxiety, hopelessness, helplessness, dread, and poor self-esteem among others, might be linked to later mild cognitive impairment (MCI), dementia, and AD, not only in a prodromal manner, but also in a causative sense.^39-42^

## Path dependence

**Theory** – The above social risk factors were addressed because they are changeable, which is essential to the premise that preventive intervention is effective. However, bringing about the change is difficult. In social science, path dependence is an established theory. One of the important aspects of this theory is the argument that an earlier choice or event largely determines the successive ones and that a change in course is difficult. Path dependence may be an unfamiliar term in health science but in order to confirm its generalizability, we provide a summary below of some of the essential points of a classic review study, a study about the standardization of the QWERTY keyboard, and two important theoretical studies.

- Once a historical choice is made, it both precludes and facilitates alternative future choices. Once a path is taken, it dictates future developments.^43^
- Important influences upon eventual outcomes can be exerted by temporally remote events. The QWERTY keyboard emerged in an effort to reduce the frequency of typebar clashes and was modified for a sales gimmick, but ever since the industry locked onto the QWERTY standard, it has been difficult to change the QWERTY’s dominance.^44^
- Particular courses of action, once introduced, can be virtually impossible to reverse. The costs of switching from one alternative to another will, in certain social contexts, increase markedly over time.^45^
- Events that take place in the early stages of an overall historical sequence are of decisive importance for the final outcome of that sequence.^46^

**Education as a critical factor** – Applying the concept of path dependence to the life course, choices or events in earlier life such as formal education in childhood, are found to largely determine situations in later life, such as occupation, income, lifestyle, and psychological status in adulthood.

Education is one of these early experiences in life. Results of some previous population-based studies have implied that education is a critical factor determining the other independent psychosocial risk factors for dementia: occupation, income, participation in social and mental activities, and psychological distress. The results of a New York study indicated that educational level and occupational attainment were strongly related,^3^ while an Australian study showed that elderly men who had worked in manual occupations had the lowest level of education,^14^ while a Canadian study stated that years of education were related to occupational complexity.^16^ The Kuopio and Joensuu survey found that individuals with low education were more likely to have a lower income level and be employed in a physical occupation or have no occupation at midlife.^18^ The U.S. AHEAD study reported that the correlation among education, income, and wealth was significant.^7^

A New Haven study revealed the correlation among fewer social connections, less education, and lower incomes.^19^ The RADC study showed a strong positive correlation between premorbid reading and education,^20^ the Religious Order Study indicated that cognitive activity had a correlation with education,^23^ and the Bronx Study revealed that level of participation in cognitive activities was associated with educational level.^24^ A survey in Japan found that high educational attainment was significantly associated with social activity level where elderly with higher education tended to be more socially active.^47^

The Chicago census reported that depressive symptoms had a negative correlation with education,^35^ and the RMAP study found that loneliness was negatively related to education.^37^

**Preliminary survey in Mexico** – Our preliminary survey in Mexico suggested that path dependency was strong in the developing world. We conducted a self-administered questionnaire survey of 440 healthy older individuals who participated in activities at the Culture Centers or Clubs of the Third Age in three cities in Mexico. These cities had similar levels of social development.^48^ The subjects participated in the survey after being informed of the purpose of the study, the confidentiality of the personal information, and voluntary nature of participation. Consent was provided by returning the questionnaire form. The overall survey is still in progress and results will be published at a later date.

Subjects were questioned on their age, gender, cohabitation status, domestic role, educational level, occupational attainment, income, frequency of individual mental activity (reading books), and participation in social activities (physical, manual, or mental activities at the Culture Centers or Clubs of the Third Age). The mean age of the respondents was 70.9 years; 85.5% were women, 26.2% lived alone, 88.1% had an active domestic role, 23.8% belonged to the low education group (less than 9 years of schooling), 84.0% had low occupational attainment (others excluding professional, administrative, and teaching careers), 35.0% had no income or received no pension, 70.1% were frequent readers, and 40.6% participated in social mental activities such as learning foreign languages, the use of computers and attending lecture.

Three scales were employed to measure the psychological distress of older people: the 17-item Philadelphia Geriatric Center Morale Scale (PGCMS),^49,50^ the 10-item Rosenberg’s Self-Esteem Scale (RSES),^51^ and the 18-item Life Satisfaction Index-A (LSI-A).^52,53^ The Spanish versions of these scales were modified for the Mexican population in cooperation with the gerontology section of the Mexican National Institute of Older Adult Persons (Instituto Nacional de las Personas Adultas Mayores: INAPAM). A composite score was created by summing the z-scores of the three scales and the first quartile defined as a low psychological status group.

Multivariate logistic regression models were used to assess the association between educational level and other possible psychosocial risk factors yielding the following results. A low level of education was significantly associated with low occupational attainment as well as with having no income after adjusting for basic confounders such as age, gender, cohabitation status, and domestic role (p<0.01). It was also significantly related to less frequent participation in mental activities after adjusting for the basic confounders, occupational attainment, and income (p<0.01). The association between a low level of education and lower psychological status remained significant even after controlling for the basic confounders, income, and mental activities (p<0.05).

## Conclusions

Education establishes a path that several psychosocial risk factors depend on. Generally, this dependency may be stronger in developing countries, because society is more severely stratified and upward social mobility is a much less frequent phenomenon for the masses than in developed countries. Upper-class or well-educated older people have more opportunities to engage in social and mental activities and may understand the value of an active lifestyle to a greater degree. Moreover, it is also more difficult to maintain a high psychological status in a stratified society.

Based on the above discussion, the importance of education should be emphasized and we suggest two general and two specific key points for the prevention of dementia in developing countries.

**Expansion in education** – The spread of basic education is indispensable. It is no easy task, but there are some successful programs such as *Oportunidades* in Mexico, providing cash payments to poor families conditioned on regular school attendance. Health scientists and organizations should support such action from a more political standpoint. The quality of education is also an important issue. Public education should include more programs that widen the choice of careers, stoke interest and develop skills in lifelong active participation in social and intellectual activities, as preventative measures against dementia for the present young and future generations.

**Targeted prevention** – In view of the limited resources for public health, the scope of preventive policy should not be universal but targeted. Resources for preventing AD, dementia, or cognitive impairment should be directed toward those with a low level of education. This is particularly appropriate because levels of education are associated with risk factors for lifestyle-related diseases,^54,55^ and these risk factors are also associated with risk of dementia.^18^

**Early and active interventions** – Due to their nature, psychosocial risk factors for dementia tend to be self-reinforcing. In order to change this path of self-reinforcement, it is necessary to implement early and active interventions. The change is difficult and becomes even more difficult over time. Besides the spread of education, the targets of early intervention should include both older people and the middle-aged, although this suggestion may be somewhat contradictory to the second point regarding narrowing the scope. These active interventions should take a “find and invite” approach toward the targeted groups, as opposed to an “open and wait” style.

**Health education** – There still seems to be a misunderstanding and lack of knowledge about dementia, especially among people with low education, because they generally have less access to quality medical information. Some people have an excessive fear of the disease, others totally reject it, and some even harbor a discriminatory attitude toward patients suffering from the disease. Others however, recognize the symptoms of dementia as a phenomena of normal aging. This environment presents a great obstacle to appropriate self-care against the disease. Self-care is indispensable in situations where there is a lack of medical service, as is the case in most developing countries. It is necessary to provide the targeted individuals with opportunities to learn about the types, clinical symptoms, pathological processes, and risk factors of dementia. Furthermore, it is also necessary for them to understand that in most cases, the disease is incurable at present, but is clinically preventable and can be controlled for a significant period of time. Dementia specialists have an important role in the efforts to disseminate dementia-related education.

**Access to activity programs** – Active participation in social and mental activities is one of the low-cost and non-risk ways in which dementia can be prevented. Some developing countries have public or community services, such as Clubs of the Third Age or for the elderly in Latin American countries, to encourage older people to participate in various activities. Generally, upper-class or wealthy people have greater access to both private and public services than the poor or disadvantaged. Most of the participants in our preliminary survey were upper-class people by national standards,^56^ although used public services for a low fee. In order to help individuals with low levels of education maintain a socially active lifestyle and ensure their continued participation in cognitively stimulating activities, it is necessary to remove the economic or psychological barriers currently faced by this group.

## References

[1] United Nations (2002). Report of the Second World Assembly on Ageing: Madrid, 8-12 April
2002.

[2] Alzheimer's Disease International (2008). The prevalence of dementia worldwide.

[3] Stern Y, Gurland B, Tatemichi TK, Tang MX, Wilder D, Mayeux R (1994). Influence of education and occupation on the incidence of
Alzheimer's disease. JAMA.

[4] De Ronchi D, Fratiglioni L, Rucci P, Paternicò A, Graziani S, Dalmonte E (1998). The effect of education on dementia occurrence in an Italian
population with middle to high socioeconomic status. Neurology.

[5] Qiu C, Bäckman L, Winblad B, Agüero-Torres H, Fratiglioni L (2001). The influence of education on clinically diagnosed dementia
incidence and mortality data from the Kungsholmen Project. Arch Neurol.

[6] Karp A, Kåreholt I, Qiu C, Bellander T, Winblad B, Fratiglioni L (2004). Relation of education and occupation-based socioeconomic status
to incident Alzheimer's disease. Am J Epidemiol.

[7] Cagney KA, Lauderdale DS (2002). Education, wealth, and cognitive function in later
life. J Gerontol B Psychol Sci Soc Sci.

[8] Lièvre A, Alley D, Crimmins EM (2008). Educational differentials in life expectancy with cognitive
impairment among the elderly in the United States. J Aging Health.

[9] Turrell G, Lynch JW, Kaplan GA (2002). Socioeconomic position across the lifecourse and cognitive
function in late middle age. J Gerontol B Psychol Sci Soc Sci.

[10] McDowell I, Xi G, Lindsay J, Tierney M (2007). Mapping the connections between education and
dementia. J Clin Exp Neuropsychol.

[11] Zhang Z, Gu D, Hayward MD (2008). Early life influences on cognitive impairment among oldest old
Chinese. J Gerontol B Psychol Sci Soc Sci.

[12] Nguyen CT, Couture MC, Alvarado BE, Zunzunegui MV (2008). Life course socioeconomic disadvantage and cognitive function
among the elderly population of seven capitals in Latin America and the
Caribbean. J Aging Health.

[13] Nitrini R, Bottino CMC, Albala C (2009). Prevalence of dementia in Latin America: a collaborative study of
population-based cohorts. Int Psychogeriatr.

[14] Jorm AF, Rodgers B, Henderson AS (1998). Occupation type as a predictor of cognitive decline and dementia
in old age. Age Ageing.

[15] Li CY, Wu SC, Sung FC (2002). Lifetime principal occupation and risk of cognitive impairment
among the elderly. Ind Health.

[16] Kröger E, Andel R, Lindsay J, Benounissa Z, Verreault R, Laurin D (2008). Is complexity of work associated with risk of dementia?: The
Canadian Study of Health and Aging. Am J Epidemiol.

[17] Bootsma-van der Wiel A, de Craen AJM, Van Exel E, Macfarlane PW, Gussekloo J, Westendorp RG (2005). Association between chronic diseases and disability in elderly
subjects with low and high income: the Leiden 85-plus Study. Eur J Public Health.

[18] Ngandu T (2006). Lifestyle-related risk factors in dementia and mild cognitive
impairment: A population-based study.

[19] Bassuk SS, Glass TA, Berkman LF (1999). Social disengagement and incident cognitive decline in
community-dwelling elderly persons. Ann Int Med.

[20] Wilson RS, Bennett DA, Gilley DW, Beckett LA, Barnes LL, Evans DA (2000). Premorbid reading activity and patterns of cognitive decline in
Alzheimer disease. Arch Neurol.

[21] Scarmeas N, Levy G, Tang MX, Manly J, Stern Y (2001). Influence of leisure activity on the incidence of Alzheimer's
disease. Neurology.

[22] Wang HX, Karp A, Winblad B, Fratiglioni L (2002). Late-life engagement in social and leisure activities is
associated with a decreased risk of dementia: a longitudinal study from the
Kungsholmen Project. Am J Epidemiol.

[23] Wilson RS, Mendes de Leon CF, Barnes LL (2002). Participation in cognitively stimulating activities and risk of
incident Alzheimer disease. JAMA.

[24] Verghese J, Lipton RB, Katz MJ (2003). Leisure activities and the risk of dementia in the
elderly. New Engl J Med.

[25] Glei DA, Landau DA, Goldman N, Chuang YL, Rodríguez G, Weinstein M (2005). Participating in social activities helps preserve cognitive
function: an analysis of a longitudinal, population-based study of the
elderly. International J Epidemiol.

[26] Katzman R (1993). Education and the prevalence of dementia and Alzheimer's
disease. Neurology.

[27] Gatz M, Svedberg P, Pedersen NL, Mortimer JA, Berg S, Johansson B (2001). Education and the risk of Alzheimer's disease: findings from the
study of dementia in Swedish twins. J Gerontol B Psychol Sci Soc Sci.

[28] Scarmeas N, Stern Y (2003). Cognitive reserve and lifestyle. J Clin Exp Neuropsychol.

[29] Richards M, Deary IJ (2005). A life course approach to cognitive reserve: a model for
cognitive aging and development?. Ann Neurol.

[30] Baldivia B, Andrade VM, Amodeo Bueno OF (2008). Contribution of education, occupation and cognitively stimulating
activities to the formation of cognitive reserve. Dement Neuropsychol.

[31] Buntinx F, Kester A, Bergers J, Knottnerus JA (1996). Is depression in elderly people followed by dementia?: a
retrospective cohort study based in general practice. Age Ageing.

[32] Geerlings MI, Schoevers RA, Beekman ATF (2000). Depression and risk of cognitive decline and Alzheimer's disease:
results of two prospective community-based studies in The
Netherlands. Br J Psychiatry.

[33] Green RC, Cupples LA, Kurz A (2003). Depression as a risk factor for Alzheimer disease: The MIRAGE
Study. Arch Neurol.

[34] Wilson RS, Evans DA, Bienias JL, Mendes de Leon CF, Schneider JÁ, Bennett DA (2003). Proneness to psychological distress is associated with risk of
Alzheimer's disease. Neurology.

[35] Wilson RS, Mendes de Leon CF, Bennett DA, Bienias JL, Evans DA (2004). Depressive symptoms and cognitive decline in a community
population of older persons. J Neurol Neurosurg Psychiatry.

[36] Karp A (2005). Psychosocial factors in relation to development of dementia in
late-life: a life course approach within the Kungsholmen Project.

[37] Wilson RS, Krueger KR, Arnold SE (2007). Loneliness and risk of Alzheimer disease. Arch Gen Psychiatry.

[38] Fernández Martínez M, Castro Flores J, Pérez de las Heras S, Mandaluniz Lekumberri A, Gordejuela Menocal M, Zarranz Imirizaldu JJ (2008). Risk factors for dementia in the epidemiological study of
Munguialde County (Basque Country, Spain). BMC Neurology.

[39] Jorm AF, Van Duijn CM, Chandra V (1991). Psychiatric history and related exposures as risk factors for
Alzheimer's disease: a collaborative re-analysis of case-control
studies. Int J Epidemiol.

[40] Mitchell AJ (2005). Depression as a risk factor for later dementia: a robust
relationship?. Age Ageing.

[41] Wright SL, Persad C (2007). Distinguishing between depression and dementia in older persons:
Neuropsychological and neuropathological correlates. J Geriatr Psychiatry Neurol.

[42] Simard M, Hudon C, van Reekum R (2009). Psychological distress and risk for dementia. Curr Psychiatry Rep.

[43] Krasner SD (1984). Approaches to the state: alternative conceptions and historical
dynamics. Comparative Politics.

[44] David PA (1985). Clio and the economics of QWERTY. Am Econ Rev.

[45] Pierson P (2000). Increasing returns, path dependence, and the study of
politics. Am Pol Sci Rev.

[46] Mahoney J (2000). Path dependence in historical sociology. Theory Soc.

[47] Aoki R, Ohno Y, Tamakoshi A (1996). Lifestyle determinants for social activity level among the
Japanese elderly. Arch Gerontol Geriatr.

[48] Consejo Nacional de Población (CONAPO) (2006). Índices de marginación, 2005.

[49] Lawton MP, Kent DP Kastenbaum R, Sherwood S (1972). The dimensions of morale. Research planning and action for the elderly: The power and potential of
social science.

[50] Lawton MP (1975). The Philadelphia Geriatric Center Morale Scale: a
revision. J Gerontol.

[51] Rosenberg M (1965). Society and the adolescent self-image.

[52] Neugarten BL, Havighurst RJ, Tobin SS (1961). The measurement of life satisfaction. J Gerontol.

[53] Adams DL (1969). Analysis of a life satisfaction index. J Gerontol.

[54] Kilander L, Nyman H, Boberg M, Lithell H (1997). Cognitive function, vascular risk factors and education: a
cross-sectional study based on a cohort of 70-year-old men. J Int Med.

[55] Kubzansky LD, Berkman LF, Glass TA, Seeman TE (1998). Is educational attainment associated with shared determinants of
health in the elderly?: findings from the MacArthur studies of successful
aging. Psychosom Med.

[56] Consejo Nacional de Población (CONAPO) (2004). Envejecimiento de la población de México: Reto del siglo
XXI.

